# Adapted diabetes complications severity index predicts dementia risk in ageing type 2 diabetes mellitus patients

**DOI:** 10.1093/braincomms/fcae079

**Published:** 2024-03-22

**Authors:** Mingyang Sun, Wan-Ming Chen, Szu-Yuan Wu, Jiaqiang Zhang

**Affiliations:** Department of Anesthesiology and Perioperative Medicine, Henan Provincial People’s Hospital, People’s Hospital of Zhengzhou University, Zhengzhou, 450052, China; Graduate Institute of Business Administration, College of Management, Fu Jen Catholic University, Taipei, 242, Taiwan; Artificial Intelligence Development Center, Fu Jen Catholic University, Taipei, 242, Taiwan; Graduate Institute of Business Administration, College of Management, Fu Jen Catholic University, Taipei, 242, Taiwan; Artificial Intelligence Development Center, Fu Jen Catholic University, Taipei, 242, Taiwan; Department of Food Nutrition and Health Biotechnology, College of Medical and Health Science, Asia University, Taichung, 413, Taiwan; Big Data Center, Lo-Hsu Medical Foundation, Lotung Poh-Ai Hospital, Yilan, 265, Taiwan; Division of Radiation Oncology, Lo-Hsu Medical Foundation, Lotung Poh-Ai Hospital, Yilan, 265, Taiwan; Department of Healthcare Administration, College of Medical and Health Science, Asia University, Taichung, 413, Taiwan; Cancer Center, Lo-Hsu Medical Foundation, Lotung Poh-Ai Hospital, Yilan, 265, Taiwan; Centers for Regional Anesthesia and Pain Medicine, Taipei Municipal Wan Fang Hospital, Taipei Medical University, Taipei, 110, Taiwan; Department of Management, College of Management, Fo Guang University, Yilan, 262, Taiwan; Department of Anesthesiology and Perioperative Medicine, Henan Provincial People’s Hospital, People’s Hospital of Zhengzhou University, Zhengzhou, 450052, China

**Keywords:** old-age, T2DM, aDCSI, diabetes-associated dementia, predictive

## Abstract

This study investigated the link between the adapted diabetes complication severity index at the time of type 2 diabetes mellitus diagnosis and diabetes-induced dementia risk in elderly patients. Elderly type 2 diabetes mellitus patients (age ≥ 60) were matched using propensity score matching. Cox regression was used to determine dementia hazard ratios; Kaplan–Meier method to assess cumulative incidence. The cohort included 256 214 elderly type 2 diabetes mellitus patients. Adapted diabetes complication severity index ≥ 1 showed higher dementia risk (adjusted hazard ratio: 1.30; 95% confidence interval: 1.27–1.34), increasing by 1.17-fold per adapted diabetes complication severity index point. Dementia risk rose progressively across adapted diabetes complication severity index scores (*P* < 0.0001). Higher adapted diabetes complication severity index scores at the time of type 2 diabetes mellitus diagnosis elevated dementia risk in elderly patients. Adapted diabetes complication severity index ≥ 1 is linked to increased dementia risk. Adapted diabetes complication severity index evaluation at the time of type 2 diabetes mellitus diagnosis could predict risk, aiding early interventions. Effective diabetes management is crucial for reducing dementia risk in this population.

## Teaser

Sun *et al*. revealed that high adapted diabetes complications severity index scores at the time of type 2 diabetes mellitus diagnosis significantly increase dementia risk in elderly patients. The risk escalates with higher scores, highlighting the importance of early assessment for timely dementia prevention and management in this population.

## Graphical Abstract

**Graphical Abstract:**
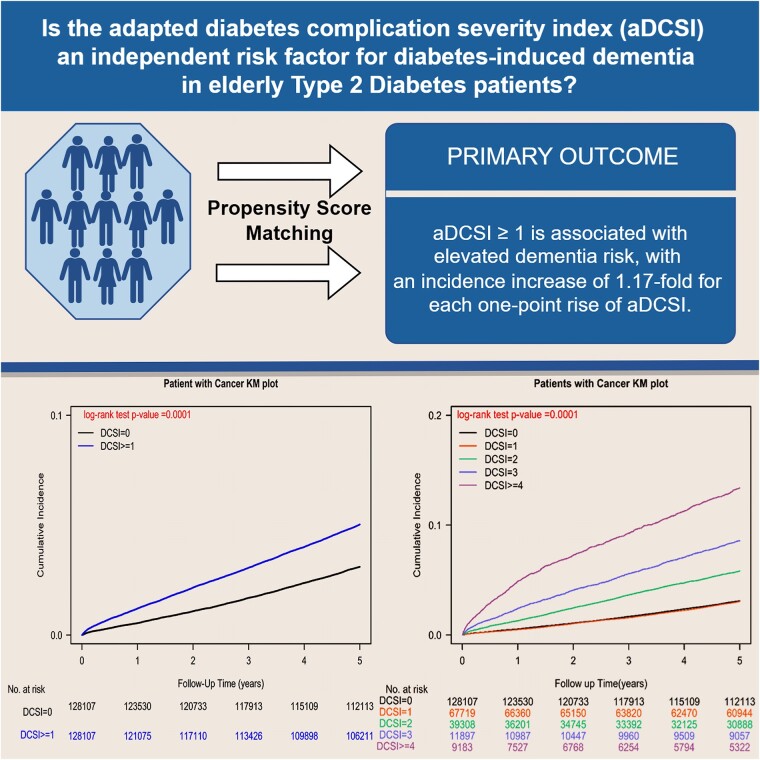

## Introduction

Diabetes-associated dementia poses a significant public health concern with widespread implications.^1^ Global estimates reveal that individuals with diabetes face a considerably higher risk of developing dementia compared to those without diabetes.^2,3^ The impact of diabetes-associated dementia extends beyond individual health, affecting national manpower and human resources.^4^ The rising prevalence of diabetes and the subsequent increase in dementia cases present challenges to healthcare systems worldwide.^4^ The financial implications of diabetes-associated dementia are substantial, encompassing healthcare utilization, medications, hospitalizations and long-term care. These costs can be overwhelming for individuals, families and healthcare systems alike.^5^ Furthermore, the indirect costs associated with lost productivity and reduced workforce participation further exacerbate the economic burden.^6^ Diabetes-induced dementia represents a significant public health concern, given the growing burden of both diabetes and dementia worldwide. With the ageing population and increasing prevalence of diabetes, the incidence of dementia is also expected to rise substantially, posing substantial challenges to healthcare systems and economies. Type 2 diabetes mellitus (T2DM) is the predominant form of diabetes, accounting for approximately 90–95% of all cases worldwide.^7^ Its high prevalence increases the population at risk for diabetes-associated dementia, amplifying the public health impact.^8^ The chronic nature of T2DM allows for potential cumulative effects on brain health and cognitive function.^9^ T2DM is closely linked to modifiable lifestyle factors such as obesity, physical inactivity and unhealthy dietary habits, contributing to the development of both T2DM and dementia.^10,11^ Thus, the quest for a simple, practical and reliable tool to predict diabetes-associated dementia in elderly T2DM patients is of paramount importance, given the substantial prevalence and potential impact on cognitive health. Such a tool would enable metabolic specialists to accurately assess patients’ dementia risk, facilitating early interventions for blood glucose control and preventive measures against dementia, promoting collaborative efforts with neurology specialists for early detection and management of dementia.

The adapted diabetes complications severity index (aDCSI) is a modified version of the diabetes complications severity index (DCSI) that omits the use of laboratory data to assess the severity of diabetes-related complications.^12^ Despite this modification, aDCSI has demonstrated strong predictive abilities for increased hospitalization, higher healthcare costs and an elevated risk of severe hypoglycaemia, as well as macro-/microvascular complications.^13^ The index provides a reliable and efficient tool for evaluating the burden of diabetes-related complications and their potential impact on patient outcomes.^12-14^ The aDCSI has shown promise as a practical and clinically useful tool to assess the severity of diabetes-related complications.^12-14^ If it can be reliably utilized to predict future dementia risk, it would become a valuable and measurable instrument for physicians. By identifying patients with a higher aDCSI score, healthcare providers can implement more proactive and tailored preventive strategies to mitigate the risk of dementia development. Such an approach would be particularly beneficial in resource allocation and planning, enabling early screening and intervention for those at higher risk, thereby potentially reducing the overall burden of dementia on healthcare systems and society.

Although aDCSI serves as an indicator reflecting certain vascular changes associated with T2DM in critical organs,^12-14^ its potential relationship with diabetes-induced dementia remains speculative, lacking substantial evidence linking aDCSI scores at the time of T2DM diagnosis to dementia risk. The extent of increased dementia risk with each incremental point in aDCSI scores remains unexplored. This information would be valuable for metabolic specialists to predict dementia risk and enable neurologists to implement preventive strategies proactively. Furthermore, public health experts could use these data to identify high-risk populations and screen them early, reducing the burden of diabetes-induced dementia for those with diabetes-related complications (aDCSI ≥ 1) at the time of T2DM diagnosis. Thus, our real-world cohort study aims to evaluate the impact of aDCSI on diabetes-induced dementia in elderly T2DM patients, contributing to informed health policy management for the nation’s welfare and well-being.

## Materials and methods

The study utilizes data from the National Health Insurance Research Database (NHIRD) outlined in the manuscript. Due to limitations imposed by Taiwan’s Personal Information Protection Act since 2012, the data is not publicly accessible in the manuscript, supplementary files or any public repository. Access requires a formal proposal approval from the relevant Taiwanese governmental department’s ethics review committee. Visit http://nhird.nhri.org.tw/en/Data_Subsets.html#S3 and https://nhird.nhri.edu.tw//en/index.html for proposal details. Informed consent requirements were waived under the Personal Information Protection Act. Dr Szu-Yuan Wu, MD, PhD, oversees the data’s integrity, with no prior presentation at scientific meetings. The study obtained approval from the Institutional Review Board of Tzu-Chi Medical Foundation (IRB109-015-B).

### Study population

This population-based cohort study utilized Taiwan’s NHIRD, a comprehensive resource containing data on disease diagnoses, underlying conditions, procedures, drug prescriptions, demographics and enrolment profiles of beneficiaries.^15-17^ Patient identifiers are encrypted to protect privacy, and linkage with Taiwan’s Death Registry ensures accurate determination of vital status and cause of death, ensuring the study’s reliability and validity.

Our study exclusively focused on elderly individuals (age ≥ 60 years) diagnosed with T2DM between 2008 and 2018, with a follow-up period extending until 31 December 2021. To ensure data accuracy, we meticulously excluded patients with missing age-related information and those with pre-existing dementia before the index date from the analysis. The case group comprised T2DM patients with aDCSI ≥ 1 at diagnosis, while the control group included individuals with aDCSI = 0 at diagnosis. Currently, there is no effective tool for predicting diabetes-associated dementia; thus, we evaluated aDCSI at T2DM diagnosis as a potential predictive tool for dementia risk. Dementia cases were classified into subgroups based on specific types, such as Alzheimer’s disease, vascular dementia or other forms of dementia. A confirmed diagnosis of dementia required a minimum of three visits within a consecutive year in NHIRD records, with validation by specialists in psychiatry, neurology or psychosomatic medicine.

### Study covariates and propensity score matching

In our study, elderly participants were categorized into distinct age groups at the index date: 60–65, 66–70, 71–75 and >75 years. To address potential confounding factors, we utilized propensity score matching (PSM) to achieve balance between aDCSI ≥ 1 and aDCSI = 0 groups, matching covariates that included age, sex, income level, urbanization level, presence of coexisting comorbidities, smoking status, presence of alcohol-related liver diseases, Charlson comorbidity index (CCI) scores and other medications (statins, anticholinergic drugs, benzodiazepines and antipsychotics; as shown in Supplementary Table 1). The index date was set 1 year after the diagnostic date of T2DM to exclude dementia occurrences within this period, which might be attributed to risk factors unrelated to diabetes. Prior to analysis, baseline matching of aDCSI ≥ 1 and aDCSI = 0 was conducted to ensure comparability of data on the index date. Duplicate comorbidities were carefully excluded from the calculation of CCI scores to prevent redundancy in multivariate analyses. Comorbidities were identified based on diagnostic codes (International Classification of Diseases, Ninth Revision, Clinical Modification, and International Classification of Diseases, Tenth Revision, Clinical Modification) recorded in in-patient records or the presence of at least two out-patient visits within a 1-year period. Comorbidities emerging within the year preceding the index date were taken into account. Continuous variables were presented using appropriate measures, such as mean ± standard deviation or median (first quartile and third quartile).

### Outcome variables

Our primary aim in this study was to evaluate the risk of dementia as the primary outcome variable. Dementia occurrence was carefully tracked from the index date until either the date of dementia diagnosis or the end of the study period, which extended until 31 December 2021. As secondary outcome measures, we examined the incidence rate (IR) and incidence rate ratio (IRR) of dementia within our cohort. To estimate the IRR of dementia, we employed Poisson regression analysis.

### aDCSI at the time of T2DM diagnosis

In this study, we aim to utilize the aDCSI at the time of T2DM diagnosis, 3 months before and after, as a primary tool to predict the risk of diabetes-induced dementia in the future. In our study, the calculation method for aDCSI was adopted from the previous study conducted by Chen and Hsiao.^13^ We will divide the patients into two groups based on the presence or absence of complications (aDCSI = 0 versus aDCSI ≥ 1) to investigate whether the severity of T2DM at diagnosis influences the risk of future dementia. By assessing aDCSI as a continuous variable and evaluating the various scores of aDCSI for diabetes-induced dementia, we aim to determine whether the severity of diabetes at diagnosis affects the subsequent risk of dementia.

### Statistical analysis

**Figure 1:**
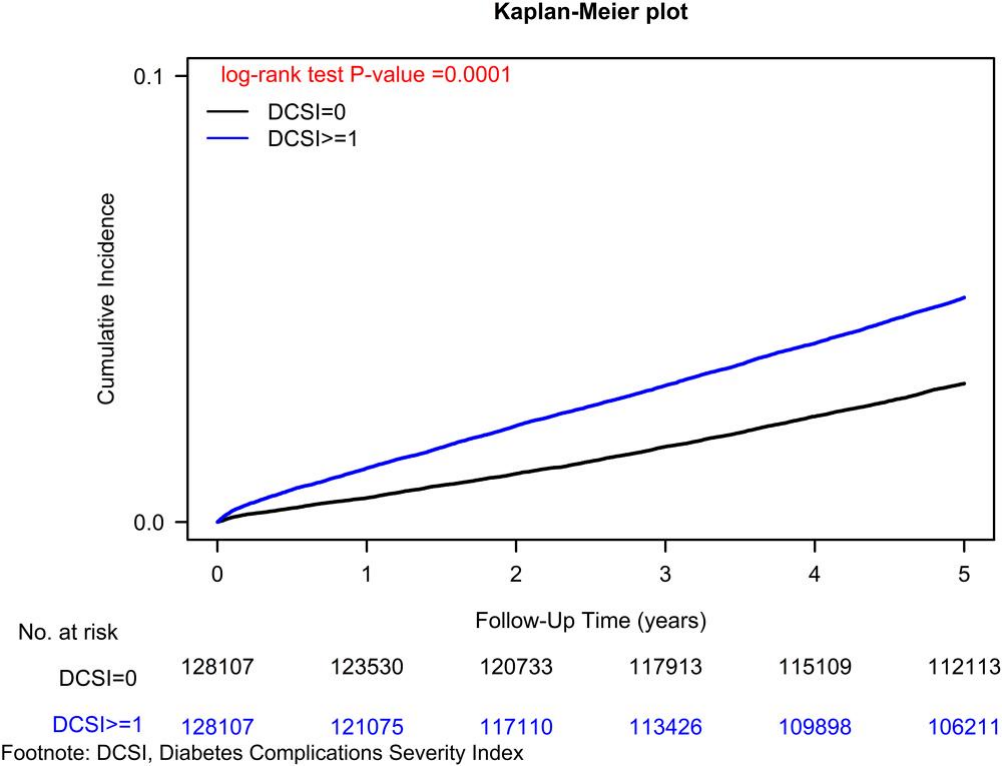
Kaplan–Meier curves for the cumulative incidence of dementia in elderly patients with T2DM stratified by aDCSI (aDCSI ≥ 1 versus 0).

**Figure 2:**
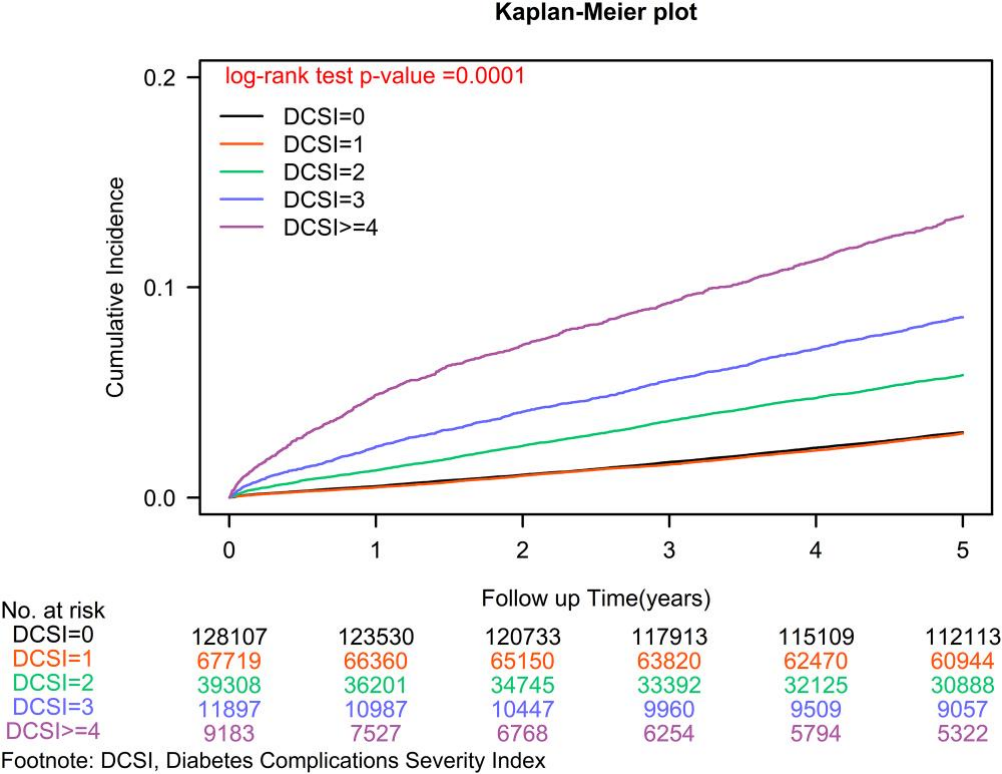
Kaplan–Meier curves for the cumulative incidence of dementia in elderly patients with T2DM stratified by varied aDCSI at diagnosis.

To comprehensively address potential confounding factors, we conducted Cox regression analysis with meticulous adjustment for a broad spectrum of covariates, including age, sex, income level, urbanization level, presence of coexisting comorbidities, smoking status, alcohol-related liver diseases, CCI scores and other medications.^18^ Additionally, we employed competing risk analysis to account for the risk of mortality. Sensitivity analyses were conducted to explore potential heterogeneity by stratifying the cohort based on baseline characteristics, ensuring consistent assessment of the impact of aDCSI = 0 versus aDCSI ≥ 1 on diabetes-induced dementia across subgroups. The cumulative incidences of dementia were assessed using the Kaplan–Meier method, and differences between aDCSI ≥ 1 and aDCSI = 0 were evaluated using a stratified log-rank test (Fig. 1). Furthermore, we estimated the incidence of dementia according to different levels of aDCSI using the Kaplan–Meier method and assessed differences using a stratified log-rank test (Fig. 2). To ensure rigorous and robust analysis of the data, all statistical analyses were conducted using SAS software (version 9.4; SAS Institute, Cary, NC, USA). The study protocols received approval from the Institutional Review Board of Tzu-Chi Medical Foundation (IRB109-015-B).

## Results

Before PSM, a total of 408 440 elderly patients with T2DM were enrolled in the study, with 215 132 and 193 308 falling into the groups of aDCSI = 0 and aDCSI ≥ 1, respectively. The aDCSI ≥ 1 group exhibited a higher prevalence of elderly individuals, lower-income levels, rural residents, coexisting comorbidities, cigarette smoking, alcohol-related liver diseases and medication usage including statins, anticholinergic drugs, benzodiazepines and antipsychotics when compared with the aDCSI = 0 T2DM patients (Supplementary Table 1). Following PSM, our study included a total of 256 214 elderly individuals in Taiwan who were diagnosed with T2DM between 2008 and 2018. The average age at T2DM diagnosis was 76.31 years for the aDCSI = 0 group and 76.91 years for the aDCSI ≥ 1 group. Through meticulous examination, we achieved a well-balanced distribution of all covariates between the two groups (aDCSI = 0 and aDCSI ≥ 1), indicating the success of our adjustment procedure in achieving covariate equivalence (as shown in Supplementary Table 1). All absolute standardized mean differences were less than 0.1, confirming the comparability of the two groups.^19^ In the non-matched covariates (aDCSI), aDCSI scores revealed the order of prevalence of diabetes complications in the aDCSI ≥ 1 group, with cardiovascular being the most common, followed by nephropathy, neuropathy, cerebrovascular, retinopathy, peripheral vascular disease and metabolic complications (least prevalent; Supplementary Table 1).

### Dementia risk and hazard ratios associated with varied diabetes severity in elderly T2DM patients

In our comprehensive analysis, we identified a significant increase in dementia risk among elderly individuals with aDCSI ≥ 1, revealing an adjusted hazard ratio (aHR) of 1.30 (95% confidence interval [CI]: 1.27–1.34; Table 1). This association was reinforced by a remarkable log-rank test result (*P* = 0.0001; Fig. 1). Furthermore, our Cox regression analysis highlighted a scores-dependent rise in dementia risk with increasing aDCSI scores. For each incremental point in aDCSI compared to aDCSI = 0, the aHR (95% CI) for dementia increased by 1.17 (1.16–1.19), signifying a greater risk of dementia with higher aDCSI scores. Additionally, our investigation of various aDCSI scores demonstrated a consistent scores–response relationship, with progressively higher aHRs for dementia observed across aDCSI scores (1.07, 1.41, 1.64 and 2.06 for aDCSI 1, 2, 3 and 4, respectively) compared to individuals with aDCSI = 0 (*P* for trend < 0.0001). These compelling findings, supported by Fig. 2 (*P* = 0.0001), further validate the scores-dependent effects of aDCSI in increasing dementia risk.

**Table 1:** Dementia risk and adjusted HRs associated with varied diabetes severity at the diagnosis of T2DM in elderly patients

Variable	Crude HR (95% CI)		*P*-value	Adjusted HR (95% CI)^a^		*P*-value
Adapted diabetes complications severity index						
aDCSI = 0	*Reference*					
aDCSI ≥ 1	1.47	(1.43–1.52)	<0.0001	1.30	(1.27–1.34)	<0.0001
Adapted diabetes complications severity index						
aDCSI as a continuous variable	1.33	(1.31–1.34)	<0.0001	1.17	(1.16–1.19)	<0.0001
Adapted diabetes complications severity index						
aDCSI = 0	*Reference*					
aDCSI = 1	1.02	(0.98–1.06)	0.3575	1.07	(1.03–1.11)	0.0004
aDCSI = 2	1.71	(1.64–1.77)	<0.0001	1.41	(1.35–1.46)	<0.0001
aDCSI = 3	2.39	(2.26–2.53)	<0.0001	1.64	(1.55–1.74)	<0.0001
aDCSI ≥ 4	3.61	(3.41–3.82)	<0.0001	2.06	(1.94–2.19)	<0.0001
*P* for trend			<0.0001			<0.0001

aDCSI, adapted diabetic complication severity index; CI, confidence interval; HR, hazard ratio. ^a^Adjusted for all covariates shown in Supplementary Table 1 using a Cox proportional regression model.

### Dementia incidence in elderly T2DM patients with varied diabetes severity

In our elderly cohort with T2DM, the incidence of dementia differed significantly between individuals with aDCSI = 0 (5.95%; 7623 individuals) and aDCSI ≥ 1 (8.40%; 10 764 individuals), with a remarkable disparity (*P* < 0.0001; Supplementary Table 1). Importantly, aDCSI ≥ 1 was associated with higher IRs of dementia compared to aDCSI = 0, as evident from Table 2. The IRR analysis further confirmed this association, showing that aDCSI ≥ 1 had an IRR (95% CI) of 1.47 (1.43–1.52) relative to aDCSI = 0. Specifically, the IR for dementia in aDCSI = 0 was 75.77 per 10 000 person-years, while aDCSI ≥ 1 had an IR of 111.49 per 10 000 person-years. Stratifying by different aDCSI scores, we observed IRRs (95% CI) for dementia compared to aDCSI = 0 of 1.02 (0.98–1.06), 1.70 (1.63–1.77), 2.38 (2.25–2.51) and 3.55 (3.35–3.75) for aDCSI = 1, 2, 3 and ≥4, respectively.

**Table 2:** Incidence rates and incidence rate ratios of dementia in elderly patients with varied diabetes severity at the diagnosis of T2DM

Variables	Events	Person-years	IR (10 000 person-years)	IRR	95% CI for IRR	*P*-value
Adapted diabetes complications severity index						
aDCSI = 0	7623	1 006 007	75.77	*Reference*		
aDCSI ≥ 1	10 764	965 448	111.49	1.47	(1.43–1.52)	<0.0001
Adapted diabetes complications severity index						
aDCSI = 0	7623	1 006 007.0	75.77	*Reference*		
aDCSI = 1	4237	547 881.2	77.33	1.02	(0.98–1.06)	0.2878
aDCSI = 2	3641	282 534.3	128.87	1.70	(1.63–1.77)	<0.0001
aDCSI = 3	1506	83 658.8	180.02	2.38	(2.25–2.51)	<0.0001
aDCSI ≥ 4	1380	51 374.1	268.62	3.55	(3.35–3.75)	<0.0001

aDCSI, adapted diabetic complication severity index; CI, confidence interval; IR, incidence rate; IRR, incidence rate ratio.

### Sensitivity analysis

In our study, we conducted sensitivity analyses to assess the impact of aDCSI ≥ 1 on dementia risk across a range of demographic and clinical factors, including age, sex, income level, urbanization level, presence of coexisting comorbidities, smoking status, alcohol-related liver diseases, CCI scores and other medications (Supplementary Table 2). These sensitivity analyses consistently demonstrated an increased risk of dementia, which is in line with the primary analysis findings. These results emphasize the robustness and generalizability of the association between aDCSI ≥ 1 and an elevated risk of dementia across various patient subgroups compared to aDCSI = 0.

### Competing risk of mortality

After meticulous adjustment for all covariates (as shown in Supplementary Table 1) using a Cox proportional regression model with competing risk of mortality (Supplementary Table 3), our analysis unveiled a noteworthy aHR of 1.27 (95% CI: 1.23–1.31) for dementia among elderly individuals with aDCSI ≥ 1. Each incremental point increase in aDCSI, in comparison to aDCSI = 0, was associated with aHR of 1.13 (95% CI: 1.11–1.14) for dementia. Moreover, we observed a significant scores-dependent increase in dementia risk across different aDCSI scores (*P* for trend < 0.0001). Specifically, the aHRs for aDCSI = 1, 2, 3 and 4 were 1.09 (1.05–1.14), 1.33 (1.27–1.38), 1.59 (1.50–1.68) and 1.63 (1.53–1.74), respectively, compared to aDCSI = 0.

## Discussion

Despite the recognized association between diabetes and dementia,^20,21^ there is a need for reliable predictive tools to identify those at higher dementia risk. Early diagnosis of T2DM and effective management may reduce the risk of diabetes-induced dementia.^22^ Our findings revealed that at the time of T2DM diagnosis, many patients had aDCSI = 0, with a relatively low future dementia IR of 75.77 per 10 000 person-years (Table 2). This highlights the importance of early diagnosis and treatment of T2DM to prevent progression and reduce dementia risk. Diabetes-induced dementia is a significant public health issue with socioeconomic implications.^1^ The lack of predictive tools calls for innovative approaches, and the aDCSI can play a valuable role (Tables 1 and 2, Figs 1 and 2). It stratifies patients by diabetes severity and dementia risk, using International Classification of Diseases (ICD) coding without lab data. In our analysis, aDCSI ≥ 1 was significantly linked to higher dementia risk in elderly individuals (aHR: 1.30, 95% CI: 1.27–1.34; Table 1), supported by a significant log-rank test result (*P* = 0.0001; Fig. 1). Each incremental point in aDCSI compared to aDCSI = 0 led to aHR of 1.17 (95% CI: 1.16–1.19). Notably, higher aDCSI scores, 1, 2, 3 and 4 (1.07, 1.41, 1.64 and 2.06, respectively), were progressively linked to higher dementia risk compared to aDCSI = 0 (*P* for trend < 0.0001; Fig. 2). These findings support the scores-dependent effects of aDCSI in raising dementia risk. By using aDCSI, physicians can proactively implement personalized strategies to mitigate dementia risk, benefiting individuals, healthcare systems and society.^21^

In our study, we found that the severity of diabetes at the time of T2DM diagnosis, as measured by aDCSI, could serve as a predictor for the development of diabetes-induced dementia (Table 1). Several factors and mechanisms may contribute to this association. One potential reason is that patients diagnosed with higher aDCSI scores (aDCSI ≥ 1) often present with diabetes-related complications, such as diabetic retinopathy, suggesting that their T2DM might have gone undetected or poorly controlled for an extended period.^23^ As a result, diabetes-related vascular changes may have progressed and affected critical organs, leading to an increased risk of diabetes-induced dementia.^24^ Furthermore, patients with higher aDCSI scores might have more severe and widespread diabetes-related vascular complications in various organs.^20,23,25^ This scenario could indicate a long-standing and uncontrolled course of T2DM, contributing to a higher probability of developing diabetes-induced dementia.^26^ It is plausible to hypothesize that the cumulative effect of diabetes-induced vascular damage on critical organs may play a significant role in dementia pathogenesis,^27-29^ underscoring the relevance of aDCSI as a valid predictor. Moreover, future research should investigate the temporal relationship between aDCSI progression and dementia onset. Longitudinal studies would provide valuable insights into how the trajectory of aDCSI over time correlates with dementia risk, shedding light on the predictive utility of aDCSI for identifying patients at higher risk of developing diabetes-induced dementia. Our study contributes to the understanding of the association between diabetes severity at T2DM diagnosis and the risk of diabetes-induced dementia. The observed link between aDCSI and dementia highlights the importance of early detection and management of T2DM to mitigate diabetes-induced complications and potentially reduce the risk of dementia. While aDCSI shows promise as a practical tool for risk prediction, further investigations are warranted to explore the multifaceted nature of dementia aetiology and its relationship with diabetes severity, leading to more effective preventive strategies and patient care in the future.

In this study, we utilized PSM to address potential confounding factors and enhance the reliability of our findings.^30,31^ PSM is a valuable statistical technique that allows us to create well-balanced comparison groups by matching individuals with similar propensities for exposure.^30,31^ This approach significantly reduces bias and ensures that any observed differences between the aDCSI = 0 and aDCSI ≥ 1 groups are primarily attributed to the varying diabetes severity, rather than confounding variables. By matching participants based on their propensity scores, we achieved a high level of covariate balance, including age, sex, income level, urbanization level, coexisting comorbidities, smoking status, alcohol-related liver diseases, CCI scores and other medications (as displayed in Supplementary Table 1). The successful matching of these covariates enhances the internal validity of our results, offering greater confidence in the accuracy of the observed association between aDCSI and dementia risk among elderly T2DM patients.^30,31^ Moreover, PSM minimizes selection bias and increases the generalizability of our findings to a broader population.^32^ The balanced distribution of confounding factors reduces the risk of biased estimates,^32^ making our results more applicable to real-world clinical settings. While PSM is a potent tool for mitigating bias, it is essential to acknowledge its limitations. The technique relies on the availability of appropriate confounding variables and assumes that all relevant confounders have been adequately accounted for. Unmeasured or unknown confounding factors may still exist,^32^ which could potentially influence the results despite our efforts to match participants based on available covariates.

Our study boasts several noteworthy strengths that lend credibility to our findings. Firstly, it is the largest investigation to date, involving 408 440 T2DM patients before PSM and 256 214 patients after PSM, providing substantial statistical power. Secondly, our research benefits from a lengthy follow-up period, with a mean duration of approximately 8 years, allowing us to observe long-term dementia outcomes accurately. Thirdly, through PSM, we effectively addressed confounding factors, achieving well-balanced comparison groups and enhancing internal validity. Additionally, our study stands as a pioneer in assessing predictive tools for diabetes-induced dementia, confirming the significance of aDCSI as a predictive factor for dementia. Furthermore, we discovered that elderly T2DM patients with aDCSI = 0 have a relatively low risk of dementia, while higher aDCSI scores exhibit a proportional increase in dementia risk with evident scores-dependent effects. Lastly, we conducted an analysis on competing risk of mortality (Supplementary Table 3) to account for potential early mortality in the aDCSI ≥ 1 group without developing dementia; however, even after considering competing risks, higher aDCSI scores remained positively associated with dementia risk. These strengths collectively support the importance of diabetes severity in predicting dementia risk, underscoring the significance of early detection and management of T2DM to mitigate potential complications, including dementia.

Despite the strengths of our study, several limitations warrant consideration. Firstly, the lack of detailed information on certain variables, such as lifestyle factors and genetic predisposition, limits our ability to fully account for all potential confounding factors. Secondly, while PSM was employed to reduce confounding, it does not entirely eliminate bias, and unmeasured or unknown confounders may still be present. Furthermore, as our study focused on elderly T2DM patients in Taiwan, the generalizability of our findings to other populations or age groups may be limited. While our study provides valuable insights into the relationship between diabetes severity and dementia risk, the aforementioned limitations should be considered when interpreting our results, emphasizing the need for further research to address these shortcomings and confirm our findings in diverse populations.

## Conclusion

Our pioneering study reveals a significant association between diabetes severity, as measured by aDCSI scores at T2DM diagnosis, and the risk of dementia in elderly patients. With the largest sample size and extensive follow-up, we robustly demonstrate that higher aDCSI scores are linked to an increased dementia risk. PSM enhances the validity of our findings. Early detection and management of T2DM are crucial, as patients with aDCSI = 0 have lower dementia risk, while elevated scores indicate higher risk.

## Supplementary material

Supplementary material is available at *Brain Communications* online.

## Supplementary Material

fcae079_Supplementary_Data

## Data Availability

The datasets supporting the study conclusions are included within this manuscript and its supplementary material.
