# Supplementary material for: Adapted diabetes complications severity index predicts dementia risk in ageing type 2 diabetes mellitus patients
Source: Brain Commun. 2024 Mar 22;6(2):fcae079. doi: 10.1093/braincomms/fcae079 (PMC10959489; doi:10.1093/braincomms/fcae079)
Supplement: fcae079_Supplementary_Data [file fcae079_supplementary_data.pdf]

Supplemental Table 1. Baseline Characteristics of Elderly Type 2 Diabetes Patients with Different Severity at Diagnosis Before and After Propensity Score Matching

	**Before Propensity Scores Matching**					**After Propensity Scores Matching**				
	**aDCSI=0**		**aDCSI≥1**		**ASMD**	**aDCSI=0**		**aDCSI≥1**		**ASMD**
	**N=215,132**		**N=193,308**			**N=128,107**		**N=128,107**		
	**N**	**%**	**N**	**%**		**N**	**%**	**N**	**%**	
**Age** (mean ± SD)	72.36 ± 13.50		76.79 ± 13.79			76.31 ± 13.24		76.97 ± 13.29		
Age, median (IQR), years-old	72.00 (63.00,76.00)		76.00 (69.00,81.00)			76.00 (67.00,85.00)		76.00 (68.00,86.00)		
Age group, years-old					0.5850					0.0980
60-65	65,472	30.43%	26,172	13.54%		28,080	21.92%	24,244	18.92%	
66-70	66,496	30.91%	46,015	23.80%		39,117	30.53%	37,917	29.60%	
71-75	45,689	21.24%	45,804	23.69%		31,095	24.27%	31,736	24.77%	
>75	37,475	17.42%	75,317	38.96%		29,815	23.27%	34,210	26.70%	
**Sex**					0.0106					0.0006
Female	103,215	47.98%	91,727	47.45%		60,222	47.01%	60,262	47.04%	
Male	111,917	52.02%	101,581	52.55%		67,885	52.99%	67,845	52.96%	
**Income level (NTD)**					0.2760					0.0390
Low income	2,513	1.17%	3,538	1.83%		1,909	1.49%	2,346	1.83%	
Financially dependent	60,664	28.20%	66,852	34.58%		39,589	30.90%	40,468	31.59%	
≤20 000	98,199	45.65%	94,769	49.02%		60,755	47.43%	60,873	47.52%	
20 001-30 000	24,318	11.30%	13,867	7.17%		12,376	9.66%	11,807	9.22%	
30 001-45 000	18,516	8.61%	9,207	4.76%		8,658	6.76%	8,099	6.32%	
>45 000	10,922	5.08%	5,075	2.63%		4,820	3.76%	4,514	3.52%	
**Urbanization level**					0.0942					0.0175
Rural	56,474	26.25%	58,941	30.49%		36,309	28.34%	37,317	29.13%	
Urban	158,658	73.75%	134,367	69.51%		91,798	71.66%	90,790	70.87%	
**Coexisting comorbidities**										
Hypertension	82,196	38.21%	121,896	63.06%	0.5131	58,991	46.05%	65,243	50.93%	0.0978

Hyperlipidemia	72,286	33.60%	74,793	38.69%	0.1061	46,415	36.23%	46,639	36.41%	0.0037
Depression	10,185	4.73%	13,748	7.11%	0.1010	7,508	5.86%	8,774	6.85%	0.0406
Anxiety	21,542	10.01%	32,275	16.70%	0.1976	16,297	12.72%	18,981	14.82%	0.0610
COPD	30,140	14.01%	50,001	25.87%	0.3002	24,137	18.84%	24,466	19.10%	0.0066
Atrial fibrillation	439	0.20%	7,636	3.95%	0.2654	439	0.34%	1,365	1.07%	0.0873
Traumatic head injury	10,553	4.91%	12,665	6.55%	0.0706	7,158	5.59%	8,042	6.28%	0.0292
Hearing loss	3,856	1.79%	6,386	3.30%	0.0960	2,888	2.25%	3,841	3.00%	0.0469
Sleep apnea	1,033	0.48%	1,149	0.59%	0.0151	668	0.52%	808	0.63%	0.0145
Liver Cirrhosis	59,237	27.54%	53,978	27.92%	0.0085	43,529	33.98%	43,088	33.63%	0.0017
Systemic Lupus Erythematosus	2,603	1.21%	3,582	1.85%	0.0522	2,028	1.58%	2,411	1.88%	0.0230
**Cigarette smoking**	26,999	12.55%	28,551	14.77%	0.0292	15,373	12.00	15,709	12.26%	0.0013
**Alcohol liver diseases**	15,296	7.11%	20,471	10.56%	0.2010	9,352	7.30%	9,411	7.35%	0.0002
**Other Medications**										
Statins	45,335	21.07%	59,317	30.69%	0.2210	32,304	25.22%	36,146	28.22%	0.0678
Anticholinergic drugs	68,175	31.69%	65,531	33.90%	0.1982	39,547	30.87%	39,602	30.91%	0.0021
Benzodiazepines	19,340	8.99%	23,042	11.92%	0.3091	10,389	8.11%	10,443	8.15%	0.0033
Antipsychotics	25,751	11.97%	25,304	13.09%	0.2914	14,105	11.01%	13,998	10.93	0.0026
**CCI Scores**										
Mean (SD)	1.05 ± 1.40		2.04 ± 1.86			1.62 ± 1.46		1.71 ± 1.78		
Median (Q1-Q3)	0.00 (0.00,2.00)		2.00 (1.00,3.00)			2.00 (0.00,2.00)		1.00 (0.00,3.00)		
CCI Scores					0.6204					0.0020
0	113,757	52.88%	46,494	24.05%		42,698	33.33%	41,571	32.45%	
≥1	101,375	47.12%	146,814	75.95%		85,409	66.67%	86,536	67.55%	
**aDCSI Score** (mean ± SD)	0.00 ± 0.00		2.00 ± 1.23			0.00 ± 0.00		1.74 ± 1.03		
	0.00 (0.00,0.00)		2.00 (1.00,2.00)			0.00 (0.00,0.00)		1.00 (1.00,2.00)		
aDCSI Score										
0	215,132	100.00%	0	0.00%		128,107	100.00%	0	0.00%	
1	0	0.00%	85,146	44.05%		0	0.00%	67,719	52.86%	

2	0	0.00%	60,925	31.52%		0	0.00%	39,308	30.68%	
3	0	0.00%	23,035	11.92%		0	0.00%	11,897	9.29%	
≥4	0	0.00%	24,202	12.52%		0	0.00%	9,183	7.17%	
aDCSI										
Retinopathy	0	0.00%	21,240	10.99%	0.4969	0	0.00%	17,121	13.36%	0.5553
Nephropathy	0	0.00%	48,732	25.21%	0.8211	0	0.00%	32,450	25.33%	0.8237
Neuropathy	0	0.00%	39,613	20.49%	0.7179	0	0.00%	29,866	23.31%	0.7797
Cerebrovascular	0	0.00%	40,111	20.75%	0.7236	0	0.00%	18,431	14.39%	0.5798
Cardiovascular	0	0.00%	103,947	53.77%	1.5252	0	0.00%	43,089	33.64%	1.1897
Peripheral vascular disease	0	0.00%	15,529	8.03%	0.4179	0	0.00%	10,894	8.50%	0.4310
Metabolic	0	0.00%	8,796	4.55%	0.3088	0	0.00%	6,950	5.43%	0.3389
**Follow-up time**					***P* Value**					***P* Value**
Mean (SD) follow-up year	8.16 ± 2.29		8.04 ± 3.11		0.7173	8.15 ± 2.50		8.04 ± 2.82		0.7061
Median (IQR) follow-up year	8.46 (7.36,9.62)		8.26 (5.51,9.36)		0.7782	8.36 (7.24,9.56)		8.25 (7.03,9.54)		0.8012
**Dementia**	10,010	4.65%	22,000	11.38%	<0.0001	7,623	5.95%	10,764	8.40%	<0.0001

Abbreviations: ASMD, absolute standardized mean differences; SD, standard deviation; IQR, interquartile range; T2DM, type 2 diabetes mellitus; CCI, Charlson comorbidity index; NTD, New Taiwan dollar; aDCSI, adapted diabetic complication severity index; N, Number.

Supplemental Table 2. Sensitivity Analyses of the Association between Diabetes Severity at Diagnosis (aDCSI≥1 versus 0) and Dementia in Elderly Patients with Type 2 Diabetes Mellitus

**Subpopulation or exposure**	**Number of patients**	**Dementia incidence**	**aHR***	**95% CI**	***P***
**Age group**, years					
60-65	52,324	871	1.24	(1.08, 1.42)	0.0023
66-70	77,034	2,005	1.41	(1.28, 1.54)	<0.0001
71-75	62,831	3,829	1.33	(1.24, 1.42)	<0.0001
>75	64,025	11,682	1.29	(1.24, 1.34)	<0.0001

**Sex**					
Female	120,484	9,775	1.26	(1.21, 1.32)	<0.0001
Male	135,730	8,612	1.35	(1.30, 1.42)	<0.0001
**Income level (NTD)**					
Low income	4,255	626	1.37	(1.16, 1.62)	0.0003
Financially dependent	121,628	9,315	1.30	(1.24, 1.35)	<0.0001
≤20 000	24,183	649	1.30	(1.10, 1.52)	0.0016
20 001-30 000	16,757	320	1.35	(1.07, 1.70)	0.0120
30 001-45 000	9,334	154	1.15	(1.03, 1.61)	0.0296
>45 000	80,057	7,323	1.30	(1.24, 1.37)	<0.0001
**Urbanization level**					
Rural	73,626	6,378	1.30	(1.23, 1.36)	<0.0001
Urban	182,588	12,009	1.31	(1.26, 1.36)	<0.0001
**CCI Score**					
0	76,581	5,406	1.27	(1.20, 1.34)	<0.0001
≥1	179,633	12,981	1.31	(1.27, 1.36)	<0.0001
**Coexisting comorbidities**					
Hypertension	124,234	11,441	1.28	(1.24, 1.33)	<0.0001
Depression	16,282	2,110	1.25	(1.14, 1.36)	<0.0001
Anxiety	35,278	3,697	1.27	(1.19, 1.36)	<0.0001
Chronic Obstructive Pulmonary Disease	48,603	5,322	1.28	(1.21, 1.35)	<0.0001
Atrial fibrillation	1,804	237	1.14	(0.84, 1.55)	0.4138
Traumatic head injury	15,200	1,714	1.32	(1.19, 1.46)	<0.0001
Hearing loss	6,729	983	1.20	(1.05, 1.37)	0.0063
Sleep apnea	1,476	102	1.68	(1.07, 2.64)	0.0240
Liver cirrhosis	79,617	5,231	1.33	(1.26, 1.41)	<0.0001
Systemic Lupus Erythematosus	4,439	499	1.29	(1.07, 1.56)	0.0086
**Cigarette smoking**	31,082	2,349	1.26	(1.14, 1.31)	<0.0001
**Alcohol liver diseases**	18,763	1,501	1.24	(1.13, 1.36)	<0.0001

**Other Medications**					
Statins	68,450	5,202	1.22	(1.11, 1.27)	<0.0001
Anticholinergic drugs	79,149	6,332	1.15	(1040, 1.26)	<0.0001
Benzodiazepines	20,832	1,562	1.16	(1.12, 1.41)	<0.0001
Antipsychotics	28,103	1,939	1.29	(1.11, 1.65)	<0.0001

Abbreviations: T2DM, type 2 diabetes mellitus; CCI, Charlson comorbidity index; NTD, New Taiwan dollar; aDCSI, adapted diabetic complication severity index; aHR, adjusted hazard ratio; HR, hazard ratio; CI, confidence interval.

*Adjusted for all covariates shown in Supplemental Table 1 using the Cox proportional regression model

Supplemental Table 3: Competing Risks Analysis of Dementia Risk and Adjusted Hazard Ratios Associated With Different Diabetes Severity at the Diagnosis of Type 2 Diabetes Mellitus in Elderly Patients

Variable	Crude HR (95% CI)		*P*	Adjusted HR (95% CI)*		*P*
**Adapted Diabetes Complications Severity Index**						
aDCSI=0	**Reference**					
aDCSI≥1	1.47	(1.43, 1.52)	<0.0001	1.27	(1.23, 1.31)	<0.0001
**Adapted Diabetes Complications Severity Index**						
aDCSI as a Continuous Variable	1.33	(1.31, 1.34)	<0.0001	1.13	(1.11, 1.14)	<0.0001
**Adapted Diabetes Complications Severity Index**						
aDCSI=0	**Reference**					
aDCSI=1	1.02	(0.98, 1.06)	0.3575	1.09	(1.05, 1.14)	<0.0001
aDCSI=2	1.71	(1.64, 1.77)	<0.0001	1.33	(1.27, 1.38)	<0.0001
aDCSI=3	2.39	(2.26, 2.53)	<0.0001	1.59	(1.5, 1.68)	<0.0001
aDCSI≥4	3.61	(3.41, 3.82)	<0.0001	1.63	(1.53, 1.74)	<0.0001
P for trend			<0.0001			<0.0001

Abbreviations: HR, hazard ratio, CI, confidence interval; aDCSI, adapted diabetic complication severity index

*Adjusted for all covariates shown in Supplemental Table 1 using a Cox proportional regression model with competing risk of mortality.
